# Genome Sequence of Border Disease Virus Strain JSLS12-01, Isolated from Sheep in China

**DOI:** 10.1128/genomeA.00502-13

**Published:** 2013-11-07

**Authors:** Xia Liu, Li Mao, Wenliang Li, Leilei Yang, Wenwen Zhang, Jianzhong Wei, Jieyuan Jiang

**Affiliations:** College of Animal Science and Technology, Anhui Agricultural University, Hefei, Anhui, Chinaa; Institute of Veterinary Medicine, Jiangsu Academy of Agricultural Sciences, Key Laboratory of Veterinary Biological Engineering and Technology, Ministry of Agriculture, National Center for Engineering Research of Veterinary Bio-products, Nanjing, Jiangsu, Chinab

## Abstract

Border disease virus (BDV) is a recognized virus in the genus *Pestivirus* and causes border disease (BD) in sheep and goats. Here, a novel BDV strain, JSLS12-01, was identified from sheep in Jiangsu Province, China. The complete coding sequence (CDS) was finished, which provides a better understanding of the molecular evolution of BDV isolates.

## GENOME ANNOUNCEMENT

Border disease (BD) in many domestic animals, mainly sheep and goats, is caused by border disease virus (BDV), a positive-sense single-stranded RNA virus in the genus *Pestivirus* of the family *Flaviviridae*, and has substantial loss-related economic implications worldwide (1). The disease is basically characterized by reproductive manifestations, such as abortions, stillbirths, barren ewes, congenital disorders of lambs, and weak lambs. BDV is confirmed to persistently infect animals (2). Severe clinical outbreaks of BD are unusual, but several epizootics have been reported in sheep (3, 4). An experimental infection carried out in pregnant ewes with BDV-4 led to a high number of stillbirths, up to 32%, and significantly reduced the body weight of lambs (5). BDV infections have been reported worldwide, such as in India, Japan, Australia, Canada, the United States, and many European countries (6). In China, BDV was first detected from some diseased goats suffering from diarrhea in 2012, and the isolates were closely grouped with the reference Gifhorn strain of BDV-3 (7).

In this study, the complete coding sequence of BDV strain JSLS12-01 was described. It was detected and identified in a sheep blood serum sample. The total RNA extracted by using the TRIzol (Invitrogen) was reverse transcribed and amplified by PCR, and PCR fragments were cloned to pJET1.2 vector (Thermo) and sequenced. The genome was assembled using DNAStar (version 7.0).

This study was performed in strict accordance with the guidelines of Jiangsu Province Animal Regulations (government decree no. 45). The protocol was approved by the Committee on the Ethics of Animal Experiments of the Institute of Veterinary Medicine, Jiangsu Academy of Agricultural Sciences (JAAS no. 20100604).

The sequenced fragment of JSLS12-01 comprising 12,227 nucleotides (nt) includes a large open reading frame of 11,694 nt, which encodes a single polyprotein flanked by near-full 5′ (278 nt) and the complete 3′ (255 nt) ends of the untranslated regions (UTRs). The 5′-UTR sequence was analyzed by ClustalX tools and MEGA 4.0.2 software with reference sequences, and phylogenetic analysis indicated that the current isolate was a newly isolated branch belonging to BDV-3 together with respective strains. Compared with the BDV strains available in GenBank, the genome sequence of JSLS12-01 has 80%, 78%, 77%, and 77% nucleotide sequence homologies with that of BDV strains Gifhorn, H2121, X818, and BD31, respectively. It displays the closest relationship to strain Gifhorn, the recognized prototype BDV-3. The amino acid sequence of the virus has 90% identity with that of Gifhorm.

BDV infection in goats occurred in China; however, no cases of BD have been reported in sheep flocks. This survey demonstrates that BDV also infected sheep in China. The results of the JSLS12-01 sequence might enhance the understanding of BDV epidemiology and evolutionary mechanisms, not only in China but also worldwide.

### Nucleotide sequence accession number.

The complete coding sequence of BDV strain JSLS12-01 has been deposited in GenBank under the accession no. KC963426.
